# Antibiotic Use in the Community: Behavioural, Contextual, and System-Level Determinants

**DOI:** 10.3390/antibiotics15060626

**Published:** 2026-06-22

**Authors:** Timo J. Lajunen, Mark J. M. Sullman

**Affiliations:** 1 Department of Psychology, Norwegian University of Science and Technology, Postboks 8900, 7491 Trondheim, Norway; 2 Department of Psychology, University of Helsinki, 00014 Helsinki, Finland; 3 Department of Social Sciences, School of Humanities and Social Sciences, University of Nicosia, P.O. Box 24005, Nicosia CY-1700, Cyprus; sullman.m@unic.ac.cy

## 1. Introduction

Antimicrobial resistance (AMR) represents a significant public health threat [1,2]. The effectiveness of antibiotics depends not only on clinical innovation but also on how these medicines are prescribed, dispensed, accessed, stored, and used in daily life. Decisions made in households, primary care centres, pharmacies, outpatient services, and veterinary settings can either support appropriate treatment or contribute to unnecessary exposure and increased selection pressure. These decisions are influenced by factors beyond knowledge, including local health systems, financial costs, access barriers, cultural practices, professional norms, regulatory enforcement, and the routine circumstances of patient care. The articles in this Special Issue show that community antibiotic use cannot be explained by individual knowledge alone. Patterns of use are shaped by social expectations, healthcare access, prescribing and dispensing practices, economic pressures, and regulatory conditions. Taken together, the contributions highlight antibiotic use as a context-dependent practice embedded in everyday routines, patient–provider interactions, and local health systems.

This Special Issue on “Antibiotic Use in the Communities, 2nd Edition” compiles 15 contributions that examine antibiotic use from varied methodological and geographical perspectives. These contributions address community-level antibiotic use, stewardship, or resistance, and together they sample cross-sectional community surveys, qualitative studies with pharmacists, analyses of prescribing and treatment pathways, a scoping review, a One Health investigation of pet owners, a hospital-based study relevant to stewardship, and a theoretical paper on interpreting resistance trends in observational research. These studies demonstrate that community antibiotic use is a context-sensitive phenomenon rather than a singular behaviour amenable to change through generic educational messages alone.

## 2. Overview of the Published Articles

### 2.1. Regional and Cultural Diversity in Awareness and Misconceptions

A primary theme across the Special Issue is regional and cultural diversity, with several contributions converging on a common paradox: awareness of antibiotic resistance frequently coexists with significant misunderstandings about how antibiotics actually work. This pattern recurs across very different populations and economic settings. In low-income urban Delhi, Rao et al. [Contribution 1] reported that many residents were familiar with antibiotics as a category of medicine but lacked detailed knowledge about AMR, with misconceptions about treating colds and influenza particularly prevalent. A strikingly similar configuration appeared in a very different European context: Pasha et al. [Contribution 2] observed high general awareness in Kosovo, yet this awareness was accompanied by confusion about resistance mechanisms and an apparent acceptability of reusing old antibiotics. The persistence of the gap among more highly educated groups is illustrated by Mora Pincay et al. [Contribution 3], who studied university students in Greater Guayaquil, Ecuador, and found that even where overall awareness was moderate (and higher among healthcare students), misconceptions about appropriate use and the meaning of resistance persisted. Iuhas et al. [Contribution 4] extended the picture into the family domain, showing that Romanian caregivers of hospitalised children typically held at least one incorrect belief about paediatric antibiotic use, even though most refrained from administering antibiotics without medical advice. Read together, these four studies suggest that public familiarity with the word “antibiotic” outpaces understanding of when and why these medicines work, reinforcing the need for public education tailored to local populations, literacy levels, and prevailing beliefs rather than delivered through generic messaging.

### 2.2. Self-Medication, Leftover Antibiotics, and the Knowledge–Practice Gap

A second theme runs in parallel with the first and helps to explain why misconceptions translate into risky behaviour: self-medication and the use of leftover antibiotics are widespread and, importantly, not confined to settings with weak knowledge. Huang et al. [Contribution 5] showed in a nationwide Chinese survey that antibiotic self-medication remained common during the COVID-19 pandemic and was patterned by demographic, behavioural, insurance, and health-related factors, suggesting that the practice is shaped by structural circumstances as much as by individual choice. Rao et al. [Contribution 1], consistent with their findings on awareness, also documented self-medication in low-income urban India, suggesting that limited detailed knowledge and self-treatment tend to co-occur. Iskandar et al. [Contribution 6] then broadened the geographic scope through a multinational study of adults from seven developing countries, who reported their experiences with leftover antibiotics. Their work makes the underlying mechanism explicit: a clear knowledge–practice gap, in which participants who could state correct principles did not consistently apply safe storage and disposal in everyday life. Pasha et al. [Contribution 2] connect back to this theme from the awareness side, having identified acceptance of reusing old antibiotics among Kosovan respondents. Read together, these studies indicate that stewardship interventions need to move past AMR awareness alone and address the practical drivers of reuse and self-treatment, including timely access to care, safe disposal pathways, proper storage advice, and the development of household routines that compete with the convenience of using what is already in the medicine cabinet.

### 2.3. Pharmacies and Healthcare Professionals: What Qualitative Inquiry Contributes

This Special Issue then examines the points at which antibiotics actually change hands, with three contributions focusing on pharmacies and healthcare professionals. Khaleel et al. [Contribution 7] used a survey of community pharmacists in southern Jordan to map the scope of the problem, documenting knowledge and practice gaps that included confusion about viral infections and the dispensing of antibiotics without prescriptions. Surveys of this kind are essential for benchmarking, but they describe what pharmacists do and know without accounting for the conditions under which those decisions are taken. Two qualitative contributions from the same research team address this gap directly, and their juxtaposition is one of the most informative features of this Special Issue.

Sullman and Lajunen [Contribution 8] conducted semi-structured qualitative interviews with 20 community pharmacists in Cyprus and used reflexive thematic analysis to interpret their accounts. The interviews moved beyond reporting rates of non-prescription dispensing to reconstructing the everyday decision environment in which pharmacists work, organised around five themes: regulation and control of dispensing; pharmacist–patient interaction and misuse; antimicrobial stewardship and public education; safety and professional responsibility; and systemic barriers. A central insight that emerged from these accounts was that pharmacists described strict adherence to prescription-only rules and treated regulation and e-prescribing as a practical “shield” that legitimised refusals and shifted the burden of saying “no” from the individual professional to the system. This kind of finding, the way regulation is experienced and used as an interactional resource at the counter, is essentially invisible to closed-format surveys.

Lajunen et al. [Contribution 9] then applied the same methodology in Brazil, where prescriptions for systemic antibiotics have been mandated since 2010/2011, yet self-medication and access without prescriptions continue. Their 20 semi-structured interviews, analysed through reflexive thematic analysis with five independent analytic cycles, surfaced themes including Access and Self-Medication, Relationships with Healthcare Professionals, and Knowledge and Beliefs about Antibiotics. In contrast to the Cypriot accounts, Brazilian pharmacists described persistent patient demand, the reuse of leftover medicines, public misunderstanding of viral illnesses, barriers to medical care, and inconsistent regulatory enforcement, all of which made refusal more costly and more ambiguous in practice. The study also foregrounded the pharmacists’ dual role as professional actors and frontline observers of public behaviour, a vantage point that allowed the interviews to capture stewardship intentions alongside the patient pressures, household practices, and inter-professional dynamics that shape what actually happens at the counter.

Reading the Cypriot and Brazilian studies side by side illustrates why qualitative inquiry is more than a complement to survey work in this field. The same professional group asked very similar questions with the same method, but produced markedly different accounts because the regulatory and structural conditions of their work differ. The interviews capture the texture of those conditions: which requests are common, how they are negotiated, when refusal is feasible, what counts as a credible reason to decline, and how pharmacists position themselves between patients, prescribers, and the regulator. Surveys can quantify outcomes such as dispensing without prescription; the qualitative work explains the mechanisms that produce or prevent that outcome and, in doing so, identifies the levers (regulatory clarity, e-prescribing infrastructure, professional backing, patient-facing communication tools) on which contextually appropriate stewardship interventions can be built. The contrast between Cyprus and Brazil also has a practical implication for the field: interventions that are effective in one regulatory environment cannot be assumed to transfer to another, and qualitative work of this kind is what makes those differences legible.

### 2.4. From Prescribing Patterns to Structured Stewardship

The contributions on prescribing and stewardship at the system level complement the pharmacy-focused work by examining what is visible in routine data and in structured programmes. Salvador Martin et al. [Contribution 10] analysed primary care antibiotic prescriptions in Castile and Leon, Spain, between 2013 and 2023, identifying temporal trends, urban–rural variation, and differences among antibiotic classes. These findings underscore the value of surveillance systems that can reveal geographic and qualitative patterns even in the absence of diagnostic linkage, and they provide the population-level context within which the pharmacy interactions described above unfold. Moving from observation to intervention, Manders et al. [Contribution 11] evaluated a structured outpatient parenteral antimicrobial therapy programme in the Netherlands and showed that mandatory involvement of infectious disease specialists, combined with an integrated workflow, was associated with shorter durations of intravenous therapy, reduced meropenem use, fewer adverse outcomes, and cost savings. Although this study addresses a more specialised pathway than the community pharmacy encounter, it reinforces the same broader point that emerged from the qualitative work: when stewardship is built into routine structures rather than left to individual decisions under pressure, antimicrobial use improves alongside care quality.

### 2.5. Extending the Lens: One Health, Hospital Exposure, and the Interpretation of Resistance Data

Several further contributions extend the focus beyond conventional adult community use and connect community antibiotic practices to wider ecological and methodological questions. Aithal et al. [Contribution 12] compared Singaporean pet owners’ knowledge and practices regarding antibiotic use for themselves and their animals, and found that knowledge and behaviours related to pet care were notably less robust than those concerning human use. This study brings the One Health framework directly into this Special Issue, recognising that veterinary antibiotic use contributes to the broader AMR ecology and that the households making decisions about human antibiotics are often the same households making decisions about their pets. Zdravkovic et al. [Contribution 13] then move into the hospital setting, examining antibiotic exposure among Serbian patients hospitalised with healthcare-associated Clostridioides difficile infection, both with and without COVID-19. Although less community-focused than the other empirical studies, this contribution shows how the cumulative effects of prior antibiotic exposure (much of which originates in community prescribing) manifest in serious clinical outcomes, and it underscores why stewardship across settings cannot be considered in isolation. Finally, Collignon and Beggs [Contribution 14] provide a methodological corrective that applies to all of the empirical work above: observed resistance in patient studies can result from multiple factors, not solely from prior antibiotic use, with heterogeneous infection profiles and confounding variables also playing significant roles. This caution informs how AMR surveillance data should be interpreted and how stewardship research, including the kinds of studies represented in this Special Issue, should be designed.

### 2.6. Synthesis and Methodological Reflection

Drawing these strands together, Ramdas et al. [Contribution 15] provide a scoping review of what community members know, believe, expect, and experience at the primary healthcare level, with particular attention to low- and middle-income countries. By organising the literature around patients, healthcare providers, health systems, and interventions, this review offers an interpretive frame for the rest of the Special Issue and reinforces the central message that inappropriate antibiotic use arises from multiple interacting factors rather than from knowledge deficits alone. Provider decisions, access constraints, regulatory enforcement, and patient expectations operate together with knowledge gaps to shape what happens in any given encounter.

The methodological breadth of this Special Issue is itself a strength, and it is most useful when each method is read for what it does best. Cross-sectional surveys provide population-level estimates of awareness, misconceptions, and reported behaviours; qualitative studies reconstruct the interpersonal and regulatory dynamics that produce those behaviours at the pharmacy counter; ecological and cohort designs clarify how prescribing and treatment pathways evolve over time; and the review and theoretical contributions situate the overall evidence base. The same variety that gives this Special Issue its breadth also limits direct comparison, because much of the evidence is self-reported, most empirical designs are cross-sectional, and populations and terminology vary across studies. The collection is therefore best read as a thematic synthesis rather than a head-to-head comparison, with the qualitative work supplying the explanatory depth that the survey and surveillance studies cannot reach on their own.

## 3. Conclusions

The articles included in this Special Issue collectively demonstrate that optimising antibiotic use within communities requires more than the dissemination of general awareness campaigns. While public knowledge remains a critical component, particularly given the persistence of misconceptions regarding viral infections, antimicrobial resistance, self-medication, and the use of leftover antibiotics, the evidence indicates that practical, contextually tailored, and system-oriented interventions are also necessary. Educational initiatives should be adapted to the cultural context and directed toward specific populations, such as parents, students, pet owners, individuals residing in low-income settings, and those who are prone to storing or reusing antibiotics. Effective pharmacy stewardship is contingent upon comprehensive professional training, the establishment of clear regulatory frameworks, the provision of robust communication resources, and streamlined access to prescriber oversight. The qualitative contributions in this Special Issue further suggest that these conditions, rather than pharmacist knowledge alone, determine whether refusal of an inappropriate request is a feasible action in any given encounter. The implementation of prescription surveillance and structured outpatient programmes complements this work by identifying problematic patterns at the population level and informing the allocation of resources.

This Special Issue offers a comprehensive and constructive synthesis of community antibiotic use across a range of settings. The findings suggest that effective prevention of antimicrobial resistance requires the alignment of public behaviour, professional practice, regulatory enforcement, health system accessibility, and data-driven stewardship. Future research would be strengthened by the inclusion of longitudinal and intervention studies, improved integration of prescribing and diagnostic data, expanded One Health approaches, further qualitative work across contrasting regulatory contexts, and greater methodological comparability across international settings. The principal conclusion emerging from these fifteen contributions is that stewardship strategies must be tailored to the specific characteristics of each community, as antibiotic use is embedded within distinct local contexts and effective interventions must be grounded in those contexts.
